# A statistical procedure to create a neighborhood socioeconomic index for health inequalities analysis

**DOI:** 10.1186/1475-9276-12-21

**Published:** 2013-03-28

**Authors:** Benoît Lalloué, Jean-Marie Monnez, Cindy Padilla, Wahida Kihal, Nolwenn Le Meur, Denis Zmirou-Navier, Séverine Deguen

**Affiliations:** 1 EHESP Rennes, Sorbonne Paris Cité, Rennes, France; 2 Inserm, UMR IRSET Institut de recherche sur la santé l’environnement et le travail - 1085, Rennes, France; 3 Lorraine University, CNRS, INRIA UMR 7502, Institut Elie Cartan, Lorraine, France; 4 UMR936 INSERM, Université de Rennes 1, Rennes, France; 5 Lorraine University, Medical School, Lorraine, France

**Keywords:** Socioeconomic status, Multidimensional index, Principal component analysis, Hierarchical classification, Small-area analysis

## Abstract

**Introduction:**

In order to study social health inequalities, contextual (or ecologic) data may constitute an appropriate alternative to individual socioeconomic characteristics. Indices can be used to summarize the multiple dimensions of the neighborhood socioeconomic status. This work proposes a statistical procedure to create a neighborhood socioeconomic index.

**Methods:**

The study setting is composed of three French urban areas. Socioeconomic data at the census block scale come from the 1999 census. Successive principal components analyses are used to select variables and create the index. Both metropolitan area-specific and global indices are tested and compared. Socioeconomic categories are drawn with hierarchical clustering as a reference to determine “optimal” thresholds able to create categories along a one-dimensional index.

**Results:**

Among the twenty variables finally selected in the index, 15 are common to the three metropolitan areas. The index explains at least 57% of the variance of these variables in each metropolitan area, with a contribution of more than 80% of the 15 common variables.

**Conclusions:**

The proposed procedure is statistically justified and robust. It can be applied to multiple geographical areas or socioeconomic variables and provides meaningful information to public health bodies. We highlight the importance of the classification method. We propose an R package in order to use this procedure.

## 

Social health inequalities are well documented in the epidemiological literature. Studies show that, for a wide array of health outcomes (infant mortality and pregnancy [1-3], cardiovascular and respiratory diseases [4-8], mental health [9,10], etc.), the burden of disease is different between deprived and affluent populations [11]. Most studies on social health inequalities use personal socioeconomic characteristics [1,6,7] which are often difficult and long to obtain, especially if an individual-based epidemiological study has to be set in order to collect them. Although contextual data cannot be used and interpreted as individual data (due among other issues to the ecological fallacy), it is easier to retrieve aggregate data from existing databases. Further, when the spatial units are small, as in our case, the ecological bias is reduced [12]. Besides, it is in some cases relevant, very convenient or even necessary to use this aggregate socioeconomic data as an alternative source of information for public health research [2,5,8-10,13-15].

Moreover, even when personal information is available, studies have shown that measures of neighborhood socioeconomic status (SES) explain significant variations in health status, even after adjustment for individual socioeconomic characteristics, suggesting that neighborhood SES may be by itself a risk factor [4,16,17].

Neighborhood SES is a complex concept involving many aspects, such as employment, income, education, housing and social bonds [18-22]. Several studies have used only one variable to represent SES, a limitation that makes it difficult to take account of the multiple dimensions of neighborhood SES. Another possibility is to study the one-by-one association (for instance with simple regression models) between the outcome of interest and different variables simultaneously with the purpose to identify if a particular component of the SES is particularly associated with the outcome. However, this comparison between associations is not trivial (for instance comparison between non nested models), especially when variables are correlated, and should be carefully conducted in order to obtain sound conclusions. Eventually, it is possible to include several socioeconomic variables in the same model but this may lead to statistical problems when dealing with multicollinearity and the large number of pmeters to be estimated.

To overcome these problems, a socioeconomic composite index may be set up at the level of a neighborhood, that may summarize the many aspects that encompasse the concept of SES with a large variety of possible techniques: additive scores with different weighting approaches (Z-score, experts weightings) [22-27], principal component analysis or factor analysis [22,26,28-31], spatial or GIS-based analyses [32,33], or others methods [34,35]. This kind of index can then be used for different purposes such as reveal the existence of social health inequalities through an epidemiological study , giving an overview of the situation for decision makers, or identify particular extreme areas where it is needed to focus public action (social planning, urban planning,…). Townsend [36] and Carstairs [24] indices, which are commonly used in various countries, cover different topics. Because they are based on only 4 variables, these indices may not always give a comprehensive representation of SES that can be used by public health bodies, at local or national level, in order to determine where action might be justified and effective. Indices that incorporate a greater number of variables from different dimensions may be more appropriate for this purpose. Moreover, these indices or Jarman’s UPA[25], were built 20 years ago and may not take into account possible modifications that occurred along time and across countries in the definitions of the variables or in the evolution of how these variables may contribute to the SES. Utilization of these indices across different countries may also be difficult due to cultural or historical distinctions (e.g. “social classes”, when defined, can have very different definitions; also, "proportion of households not owner of their house" vary considerably across countries); comparisons within countries may also be hampered by demographic and urban policy factors (e.g. the "proportion of households not owner of a car" highly depend on the availability of public transport and sharply contrast central urban areas and peri-urban or rural areas). Moreover, regardless of the creation procedure, the interpretation of the variables included in an index can be very different according to the implementation area. For instance, variables like ratio of individual houses or proportion of farmers do not have the same interpretation from a SES point of view in urban or rural areas. The interpretation of each variable included in an index must of course be done according to the context. In this setting, it could be helpful to have a versatile procedure which would allow taking into account these changes and selecting variables among a given set rather than fixing a mandatory list of variables.

A rigorous methodological approach is required to ensure that the index is statistically well founded and provides a good approximation of SES. Principal component analysis appears to be particularly suitable for developing composite indices because this statistical method creates non-correlated linear combinations of the variables with maximal variance, which allows the best contrast between statistical units. Furthermore, in ecologic epidemiological studies, mapping is a crucial step for showing the spatial distribution of deprivation in public health studies. While discretization of quantitative variables is frequently used for this purpose, mainly by using quantiles, it is an arbitrary technique which relies on the categories sample sizes rather than on similarities between units. Yet several other methods, such as hierarchical classification, are available to create homogeneous categories of similar spatial units.

In this context, this study presents a procedure based on statistical criteria and justification for selecting socioeconomic variables in order to create a neighborhood socioeconomic index meant to provide meaningful information to public health bodies and allow epidemiological assessment of social health inequalities. In view to assess its ability to be generalized, the procedure was applied in three contrasted French metropolitan areas to create both area-specific and global socioeconomic indices. Eventually, an R package was created in order to give an easy way to implement the procedure in a variety of contexts. This package contains the basic functions needed to run the procedure, obtain the corresponding SES index and create categories.

## Material and methods

### Study setting and small area level

The study was carried out in three large metropolitan areas in France: Lille (Nord Pas de Calais region, northern France), Lyon (Rhône-Alpes region, central and eastern France) and Marseille (Provence-Alpes-Côte d’Azur region, south eastern France) with a total population of around 3.8 million. These urban areas are the three largest in France after Paris. They differ in some important socio-demographic features. For instance, the Lille Métropole has a higher population of people under age 25, more blue-collar workers and individual houses than the other cities; Grand Lyon has a higher rate of foreigners and white collar workers, and fewer people with no qualifications; while the Aix-Marseille urban unit has a higher rate of single parent families, higher unemployment, a lower rate of people with steady jobs and less social housing (see in Additional file 1 the detailed socioeconomic characteristics of the metropolitan areas).

The statistical units were the sub-municipal French census block groups (called IRIS for “Îlots Regroupés pour l’Information Statistique”) defined by the National Institute of Statistics and Economic Studies (INSEE). These are the smallest units for which socioeconomic and demographic information is available from the French national census (Table 1). These units have an average of 2,000 inhabitants and are constructed in collaboration with local actors (municipalities and communities) to be as homogeneous as possible in terms of socio-demographic characteristics and land use. They also take account of physical obstacles that may break up urban landscapes, such as arterial roads, green spaces, bodies of water, and must have unambiguously identifiable contours, stable over time. Census block groups (BGs) are divided into three distinct categories in order to take into account some special cases: 1) “housing” BGs represent the vast majority of BGs (92% for all France in 2008) and count generally between 1800 and 5000 inhabitants; 2) “activity” BGs include at least 1,000 employees and at least twice as many employees as residents (e.g. industrial estates or business districts); and 3) “miscellaneous” BGs are specific wide areas sparsely populated (leisure parks, port areas, forest, etc.). As activity and miscellaneous BGs have some particular profiles due to the way they are defined, this study only considered housing BGs for the creation of the socioeconomic index. Housing BGs were treated in our statistical analysis as active units while activity and miscellaneous BGs were treated as supplementary units (meaning that they were not part of the construction of the socioeconomic index but will have an index value).

**Table 1:** Description of the three study urban areas

	**Lille Métropole**	**Grand Lyon**	**Aix-Marseille**
Population in 1999 (inhabitants)	1,091,438	1,193,384	1,349,772
Population in 2007 (inhabitants)	1,106,885	1,260,348	1,434,845
Number of municipalities	85	58	38
Number of census block groups	506	510	630
Number of housing blocks (% of census block groups)	475 (94%)	465 (91%)	563 (89%)
Number of census block groups with missing median income (% of census blocks)	119 (24%)	95 (19%)	106 (17%)
Area (km^2^)	611.45	527.15	1289.59

### Socioeconomic data

Socioeconomic data were taken from the 1999 national census (provided by the INSEE) and provided counts of population, households and residences at BG scale covering all the social, economic and demographic aspects. Median income (for 2001) at the BG scale was taken from another national study also provided by the INSEE (“Revenus fiscaux des ménages”, INSEE – DGI). Using this raw data, 48 variables were defined at the BG scale based on the INSEE definitions. These variables were chosen to be representative of the theoretical concepts of SES and in line with the variables most often used in the literature [21,22,24-31,33,36,37]. We also introduced some variables which were not totally part of the SES concept but that could be considered as linked with it (and could also give insight about the neighborhood environment). For instance we included the proportion of people over the age of 65, which could reflect the proportion of retired people; or the proportion of people who have moved from their municipality since the last census which could give indications about the “residential instability” of the neighborhood population.

All variables were associated with family structure, household type, immigration status, mobility, employment, income, education and housing (a detailed list of these variables can be found in Table 2). The aim of introducing a spectrum of variables broader than just the variables known to be indicators of the SES was to offer the possibility to examine the utility of taking into account some “proxies” and also to have a data driven approach in order to determine the variables which maximize the index’s variance.

**Table 2:** Description of the first selection of 48 socioeconomic variables at the census block group scale

***Unless stated otherwise, variables are proportions expressed in %***		**Var. Id.**
**Family and household**	People under the age of 25 in the total population	1
	People over the age of 65 in the total population	2
	People living outside the household (boarder students, soldier in garrison, people in jail, people in nursing home or in hospital, etc.) in the total population	3
	SINGLE-PARENT FAMILIES IN THE TOTAL POPULATION	4
	Householders living alone in the total population	5
**Immigration and mobility**	FOREIGN PEOPLE IN THE TOTAL POPULATION	6
	FOREIGN IMMIGRANTS (SINCE THE LAST CENSUS) IN THE TOTAL POPULATION	7
	People who have moved from their municipality since the last census in the total population	8
**Employment and income**	*People in the labor force in the total population*^a^	9
	Men in the labor force in the total male population ^a^	10
	Women in the labor force in the total female population ^a^	11
	*Unemployed people in the labor force*^b^	12
	Unemployed foreigners in the labor force ^b^	13
	Unemployed people in the 15-24 years old labor force ^b^	14
	Over 50 years old unemployed people in the labor force ^b^	15
	Unemployed people in the male labor force ^b^	16
	Unemployed people in the female labor force ^b^	17
	People unemployed for more than 1 year in the labor force ^b^	18
	SELF-EMPLOYED (INDEPENDENT WORKERS, EMPLOYERS, ETC.) IN THE LABOR FORCE	19
	PEOPLE WITH UNSTABLE JOBS IN THE LABOR FORCE (APPRENTICES, TRAINEES, TEMPORARY JOBS, ETC.)	20
	PEOPLE WITH STEADY JOBS IN THE LABOR FORCE	21
	Farmers in the labor force	22
	Managers in the labor force	23
	Blue-collar workers in the labor force	24
	MEDIAN INCOME PER CONSUMPTION UNIT (IN EUROS PER YEAR)^c^	25
**Education**	People 6-15 years old attending school in the 6-15 years old population	26
	PEOPLE WITH NO SCHOOL GRADUATION (AND NOT STUDYING) IN THE 15 YEARS OLD AND MORE POPULATION	27
	PEOPLE WITH BASIC OR INTERMEDIATE GENERAL OR VOCATION QUALIFICATIONS (AND NOT STUDYING) IN THE 15 YEARS OLD AND MORE POPULATION	28
	PEOPLE WITH GENERAL OR VOCATIONAL MATURITY CERTIFICATES (AND NOT STUDYING) IN THE 15 YEARS OLD AND MORE POPULATION	29
	People with at least a lower tertiary education (and not studying) in the 15 years old and more population	30
	People with a higher educational degree (and not studying) in the 15 years old and more population	31
	Students in the 15 years old and more population	32
**Housing**	Individual houses in the main residences	33
	Multiple dwelling units in the main residences	34
	NON-OWNER-OCCUPIED IN THE MAIN RESIDENCES	35
	Subsidized housing in the main residences	36
	Main residences built before 1968	37
	Main residences built after 1990	38
	Main residences less than 40 m^2^	39
	Main residences larger than 150 m^2^	40
	Main residences without bathtub or shower	41
	Main residences without toilet	42
	Main residences without heating	43
	Main residences with a parking space (garage or other)	44
	MAIN RESIDENCES WITH MORE THAN ONE PERSON PER ROOM	45
	AVERAGE NUMBER OF PEOPLE PER ROOM^c^	46
	HOUSEHOLDS WITHOUT A CAR	47
	HOUSEHOLDS WITH 2 OR MORE CARS	48

^a^Redundant group “labor force”.

^b^Redundant group “unemployment”.

^c^Not a proportion.

UPPERCASE : variables selected commonly for each metropolitan areas and global analysis.

*Italic*: variables selected during the “reduction of redundant groups” step for the global analysis.

Some of the variables were intentionally redundant and represented the same notion, in view to determine which best represented this notion. There were two such groups: 7 variables of unemployment (ID 12 to ID 18 in Table 2) and 3 variables of labor force (ID 9 to ID 11 in Table 2). There were an unexpectedly high number of missing values for median income (see Table 1) but, willing to keep this variable in the analysis, we filled missing values with the average value of the adjacent BGs.

### Creation of the socioeconomic index

**Figure 1:**
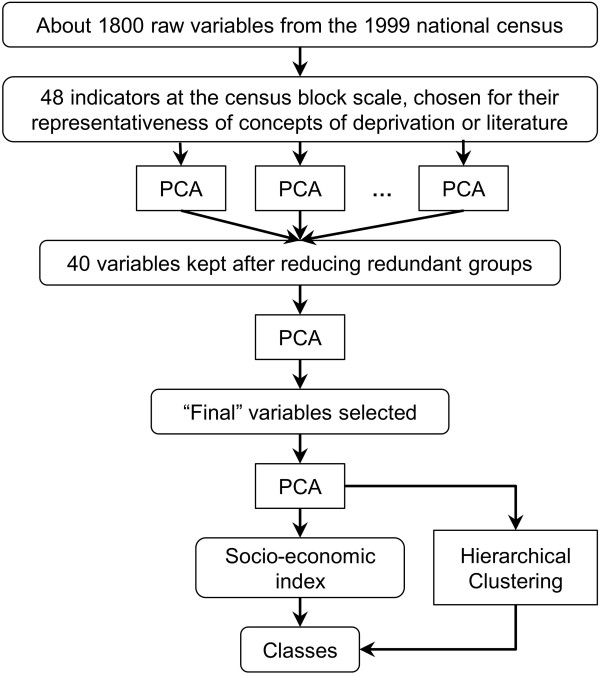
Steps of the socioeconomic index creation.

The socioeconomic index was created by improving and expanding a procedure previously developed by our team [38]. The three steps (Figure 1) described below were used:

– *Step 1 - Study of the redundant variables* (in our case variables ID 9-11 and ID 12-18 aforementioned). To avoid issues due to redundant covariates (correlation over 0.8 for most of them in all the study areas) one variable was selected from each group by applying principal component analysis (PCA, see Additional file 2) to each of the two groups of redundant variables. The first component was a good representation of the group of variables, strongly correlated with all of the variables (if not and if a variable was not well represented by the first component, - a situation that did not happen in our case - this variable should actually not have been part of the redundant group and should not have been included in it). However, to ease interpretation, the variable with the largest correlation with the first component was selected. Reducing the two redundant groups to one variable each reduced, in our case, the number of variables to 40 (the two selected variables at this step are shown in italic font in Table 2).

– *Step 2 - Selection of the variables*. PCA on these 40 variables (i.e. after selection of one variable per group of redundant variables) was used to select the variables with a contribution to the first component larger than the average one, i.e. variables that were best correlated with the first component.

*Step 3-Construction of the final index*. A final PCA was carried out including the variables selected in step 2. Provided that the first component of this PCA could be interpreted (according to the meaning of the variables in the given context) as a “SES component” (which was expected giving the variables selected and confirmed *a posteriori*), it was used to calculate the socioeconomic index as the reduced first component. This normalization gave an index with mean 0 and standard deviation 1. Since the purpose of the whole procedure was to create a single index, the second and subsequent components of the final PCA were not used (in practice, the variance explained by the second component was far below that explained by the first component and there was no clear interpretation of it).

The choice of PCA as the main technique in the procedure was done in order to use as little constraints and hypotheses as possible, as well as to keep a strongly data driven approach. This approach is not based on a model set a priori, like Factor Analysis. Moreover, it is known that a PCA where the last eigenvalues are near and close to 0 gives results very similar to those of a Factor Analysis.

This procedure was applied to each metropolitan area independently (giving socioeconomic indices specific to each area) and to the three urban areas altogether. This produced four different indices that, as a result, could be compared. Step 1 was kept in the procedure and applied to each metropolitan area because differences between them in their socioeconomic make-up might lead to different choices.

### Hierarchical clustering and optimal thresholds

Socioeconomic categories were created so that the index could be used as an explanatory variable to determine possible non-linear relationships in a variety of applications, and be used for mapping.

Defining socioeconomic index quantiles is a common technique but it is sometimes unsatisfactory. Indeed, classes created with quantiles are only based on the number of units and therefore may not correctly classify units according to their similarity if they are not distributed homogeneously. This can lead to merging in the same class very different groups of units, or to split into two classes a homogeneous group.

Hierarchical clustering (HC) is frequently used after PCA [39] in data mining to create meaningful categories. Given a set of *p* variables measured on *n* elements, each element is represented as a point in ℝ^p^. A distance between elements *d* (usually the Euclidian distance) and a distance between categories *Δ* (based on *d*) are defined. HC algorithm creates a hierarchy of categories step by step by merging at each step the two categories which are the nearest according to *Δ*. When *Δ* is a particular distance (the Ward’s distance), this algorithm allows to obtain categories homogeneous in their composition and heterogeneous between them (i.e. with a maximum between-categories inertia). The most appropriate partition is then selected from the hierarchy of categories. More methodological details about HC are available in Appendix 2.

However, HC is a multidimensional technique which uses several components of a PCA (often 5 or 10). Now, we wanted here to create the categories from a one-dimensional index (it is also possible, but not for the same purpose, to keep directly the categories created by HC in order to have a qualitative index). Then, we used HC as a reference to determine “optimal” thresholds able to construct a one-dimensional classification as close as possible to the HC. There were two possible cases, depending on the number of categories:

either the categories constructed with HC were not distributed along the first principal axis of the PCA (the second and subsequent axes affecting classification) in which case it was not possible to determine thresholds along the first axis that would be able to correctly approximate HC categories. The index could not be used by itself and the number of categories was therefore reduced.

Or the categories were distributed along the first component of the PCA (i.e. our socioeconomic index). In this case, optimal thresholds were determined using a simple iterative algorithm: at each step, categories were defined with new thresholds along the index values and the concordance rate between this classification and the clustering using HC were calculated. Only values with the best concordance percentage were kept. It created socioeconomic categories using the socioeconomic index by itself.

### Comparison of indices and classifications

Pearson’s coefficient of correlation was used to compare the area-specific indices to the global one, which encompasses the 3 metropolitan areas, and also the Carstairs’ and Tonwsend indices with ours.

Carstairs’ index [24] was constructed as the sum of the standardized proportions of total unemployment, of households without a car, of households with more than one person per room, and of blue-collar workers (since French census do not use “social classes”). Townsend’s index [36] was constructed as the sum of the standardized log-proportions of total unemployment and households with more than one person per room, and the proportions of households without a car, and of non-owner-occupied main residences.

The concordance percentage was used to compare pairs of classifications. This is the percentage of BGs in the same class in both classifications (the diagonal of the confusion matrix). The R software [40], with the FactoMineR package [39] and the SesIndexCreatoR package, was used to create the indices and clustering, to determine thresholds and draw comparisons.

### R package

Since the procedure described here is more complex than for some other SES indices, we specifically developed the SesIndexCreatoR package. The version 1.0 of this package (currently freely available on the website of the Equit’Area project: http://www.equitarea.org/documents/packages_1.0-0/) contains the basic functions needed to run the procedure (in its entirety or only in some steps) and to obtain the corresponding SES index. The user may also create categories of this index with different methods (hierarchical clustering with or without k-nearest neighbors, quantiles, or intervals). We project to extend the package in the future and among other improvements we foresee to add tools to help the interpretation of these categories and the visualization of the results.

## Results

### Constructing indices, selecting variables and determining contributions

**Figure 2:**
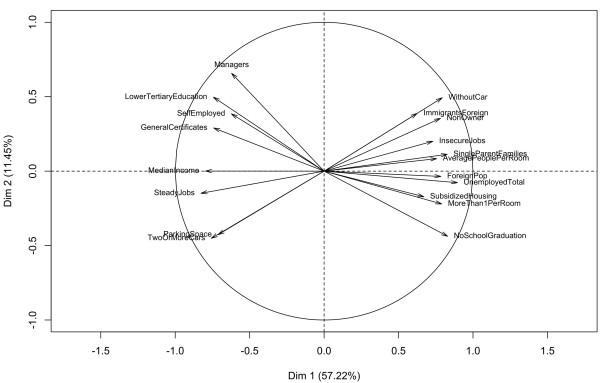
Circle of correlation for final step, global analysis.

The four socioeconomic indices were built as the first component of PCA using the data for each urban area septely and for an overall analysis of the combined sets of data. In each case, this first component of the PCA was positively correlated with variables of low SES (unemployment, single-parent families, overcrowding, etc.) and negatively correlated with variables of high SES (income, steady jobs, high level of education, etc.); therefore, it was interpreted as a true SES component (see Figure 2). It always explained a large proportion of the total variance (Table 3). By contrast, the second component had never a clear interpretation and explained less than 17% of the total variance.

**Table 3:** Percentage of variance explained by the two first components of the final PCA, by area

	**Lille Métropole**	**Grand Lyon**	**Marseille urban unit**	**Global**
1^st^ component	60.73%	57.79%	57.29%	57.22%
2^nd^ component	12.13%	16.71%	14.66%	11.45%

Fifteen of the 20 variables selected by the procedure (step 1 to 3) were common to all four indices and accounted for more than 77% of the construction of the first component (see below). This result showed that the procedure was robust and that, despite the substantial socio-demographic differences between the urban areas, the same common variables could explain a large part of the socioeconomic variability. Now, the ranks of the contributions of the variables to the index were different in each urban area (Additional file 3) although none was far from the overall average contribution.

For Lille Métropole, the procedure selected 21 variables for the index (for Grand Lyon, Marseille urban area and global analysis it selected 20, 20 and 19, respectively). The first component of the final PCA explained 61% (resp. 58%, 57% and 57%) of the total variance while the second 12% (resp. 17%, 15% and 11%). The variables common to all four indices contributed 80% (resp. 84%, 78% and 88%) to the index. In each metropolitan area, local experts associated with the project confirmed that our SES index globally well-represent the socioeconomic true profile of the neighborhoods.

### Comparisons between indices

The indices in each metropolitan area were compared with the global index restricted respectively to the BGs of each area (the global index was constructed on the BGs of the three areas altogether but, to allow comparison with the area-specific index, only BGs of this area were considered), as well as with those proposed by Carstairs and Townsend.

In general, all the correlations between the area-specific and the overall index restricted to the BGs of each metropolitan area were above 0.9 (Table 4) with a clear linear association (Additional files 4, 5 and 6). There were very good correlations between our indices (constructed either for each urban area or for the three areas altogether) and the Carstairs and Townsend indices (always larger than 0.91) suggesting that the socioeconomic dimension measured by our index is very close to that measured by the well-known and often used Carstairs and Townsend indices.

**Table 4:** Correlation coefficients between indices

	**Lille Métropole**	**Grand Lyon**	**Marseille urban unit**	**Global**
Global ^a^	0.99	1	0.99	/
Carstairs	0.92	0.96	0.91	0.94
Townsend	0.98	0.94	0.96	0.96

^a^When comparing the global index with a city index, global index is restricted to that particular city’s census block groups.

### Comparisons between classifications

The initial number of categories we tested for classification was five because it is a usual number of categories used in spatial epidemiology, especially for mapping. For all four indices, the categories obtained through HC depended both on the first and second axes of the PCA and the categories were not distributed solely along the first component. This observation suggests that 5 categories were too many should the objective be to use only the first component (i.e. our index). The analysis showed that classification with quintiles was not optimal, since the hierarchical clustering gave very different results. The number of categories was, therefore, reduced until categories could be constructed only using the first axis of the final PCA. In each case, the largest number of categories was three. This number creates the optimal classification according to the HC. Average values of the common variables for the global analysis can be seen in Additional file 7.

Concordance rates between the different techniques are shown in Table 5. Very low concordance was found for the HC with 5 categories and quintile classifications, with less than 50% of similar classifications for the three metropolitan areas and about 60% for the metropolitan areas taken together. HC was unable to construct 5 categories using only the first PCA axis. The concordance rate between HC with 3 categories and terciles was between 69% and 78%. This could be explained by the smaller number of categories. Even such concordance rates mean that about one quarter of the BGs had a different class across the two classification methods. When comparing the SES index categories (created by HC or by optimal threshold) with Carstairs’ and Townsend’s indices categorized through quantiles, the concordance rates remained low.

**Table 5:** Concordance rates between different clustering techniques and between indexes

	**HC (5) **^**a **^**vs. quintiles **^**b**^	**HC (3) **^**a **^**vs. tiertiles **^**b**^	**HC (3) **^**a **^**vs. optimal thresholds **^**b**^	**Optimal thresholds vs. tiertiles **^**b**^	**Carstairs**		**Townsend**	
					**HC (5) **^**a **^**vs. quintiles **^**b**^	**Optimal thresholds vs. tiertiles **^**b**^	**HC (5) **^**a **^**vs. quintiles **^**b**^	**Optimal thresholds vs. tiertiles **^**b**^
Lille Métropole	41%	78%	98%	79%	38%	70%	42%	78%
Grand Lyon	48%	74%	93%	78%	47%	77%	40%	75%
Marseille urban unit	48%	69%	97%	67%	51%	67%	50%	69%
Global	63%	71%	97%	72%	57%	70%	55%	71%

^a^Hierarchical Clustering using Principal Components (in parenthesis, the number of categories chosen).

^b^Concordance rate (percent of census block groups categorized into the same class using the two different clustering schemes).

By contrast, the concordance rates between HC in 3 categories and optimal thresholds were between 93% and 97%, confirming that the classification in three categories was fully along the first axis. Additional files 8, 9 and 10 show for each metropolitan area maps of the three socioeconomic categories created either by tiertiles or optimal thresholds, and the range of the SES index for each category (the higher the index, the most deprived the area).

## Discussion

This study developed a rigorous statistical procedure to create socioeconomic indices in urban contexts, improving and extending a previous work.[38] The procedure was applied and validated on three different French urban areas and proved its robustness in different socio-demographic settings. An R package was developed in order to help applying this procedure in other contexts.

As for most studies developing new methodologies to construct a neighborhood socioeconomic index, the preliminary selection of variables was based on a literature review [10,15,26,27,29]. The social and material deprivation index developed by Pampalon et al [28] included people without qualifications, employment ratio, average income, individuals living alone, individuals divorced, septed or widowed, and single parent families. They chose these variables according to four criteria: well documented health links, variables previously used as “geographic proxies” in social health inequality studies, variables belonging to the material or social dimension of deprivation and the availability of data for their study area. Carstairs and Townsend followed a similar procedure for selecting 4 variables characterizing neighborhood deprivation in their indices. However, this approach was only a preliminary step in the construction of our index. One originality of our procedure lays in selecting the final variables for the index by usage of data mining techniques rather than only information gleaned from a literature review, allowing to discard part of the subjectivity that may influence the choice of the variables. This data driven approach allows the data “speak by themself”. Although it was what we expected, it was not sure, before the PCA was implemented that the first component would be a good socioeconomic index. This appeared *a posteriori* as the PCA explored the data and revealed their underlying structure.

About 20 variables, a number not defined *a priori*, were selected for each metropolitan area, encompassing the various domains of SES. This allowed to determine the common determinants of SES in the various areas and also to select determinants which are more specific in each area. The larger number of variables compared with other indices gives room for a finer spatial description of SES and of specific characteristics of each metropolitan area, providing information which public health bodies might find helpful in determining key targets for local actions. Indeed, once the index is constructed and used to identify BGs with the lowest SES, it is possible to return to the variables that compose the index in order to see which ones could be a leverage for action, a property that more simple indexes lack. Using this method (use such an index, in a quantitative or qualitative way, to identify lowest SES areas and then go back to the individual variables to have more details) in an epidemiological study to describe the spatial distribution of some disease or cause of mortality in a metropolitan area will not only allow to flag communities where the risk is highest, but will also provide information on the social and economic characteristics of these communities upon which appropriate and focused preventive policies can be devised and implemented.

The large number of common variables (15 of the 20 variables) across the metropolitan areas shows the stability of the results and the good representation of the underlying concept of SES conveyed by the index. These variables reveal the common determinants of SES in different French metropolitan areas, at BG level, which is the smallest administrative unit for which census data is available. The specific SES patterns in each area can be assessed in two different ways: through the variables which are specific to each area, and through the relative contribution of each variable to the final index. As a result, the procedure proposed in this study can be used alternatively to build a city-specific index which can be applied locally, for instance to determine priority BGs for local action, or a global index to compare a set of cities with the same metric.

However, one should remember that data and indices used here are area-based and not person-based. Indeed, although BGs are constructed in order to be as homogeneous as possible, there is still individual variability within them which cannot be assessed by aggregated data. Therefore, as it is now well-known, inference at the individual scale from indices created at the BG scale can be tricky due to the ecological fallacy. SES indices presented here are neighborhood SES indices and should be used as a way to assess the contextual socioeconomic setting in which people live rather than a way to approximate the individual SES.

When socioeconomic indices were first constructed, categories were delineated to show the spatial distribution of SES on maps and to investigate the existence of non-linear social relationships with some outcome of interest. So far, to our knowledge, most of the studies classifying deprivation scales have used quantiles [2,10,13,15,27,29,33] without questioning the validity of this classification method from a statistical point of view. This simple approach should be used with caution; our study suggests that it might put dissimilar geographical units in the same class and septe similar units, according to HC.

Using HC, the first dimension alone of the final PCA was not sufficient to create 5 socioeconomic categories. Although we could have kept the results of the HC as a qualitative index, this would have contradicted with our aim to have a one-dimensional index. In this study, but without possible generalization to other data, it was preferable to use a 3-categories classification built only with the first component of the final PCA.

Despite its statistical justification, this study has some limitations. Some are induced by the very nature of an index. Since indices are composite syntheses of several variables, they have no unit. This can reduce the interpretability of their application, especially regression models, the meaning of an increase or decrease of one unit of the SES index being difficult to express. From a public policy point of view, an index alone cannot give indications on how to operate to change the situation. Although the indices created by the procedure we propose share these limitations, we think they are interesting as first indicators of ‘global’ neighborhood SES and as a synthetic tool to point out the situation to policy makers. Eventually, one may return, as aforementioned, to the variables composing the index to have a better insight of the actual situation of the identified neighborhoods and the variables that most contribute to this signal.

Secondly, median income had to be estimated where the data was missing. Because BGs with incomplete information on median income were a minority (maximum 24% for the Lille metropolitan area) and because only one variable among the 20 used in the indices had such missing data, incompleteness has probably little effect. A perspective for improvement could be to use more advanced techniques to handle missing data.

Thirdly, utilization of a large amount of data requires preption and calculation before applying the procedure, which is time consuming. It also calls for technical know-how. This procedure is clearly more complex than number of other indices. We think this is the price to pay for a deeper analysis of SES and its determinants and a more detailed interpretation of the results. While our index showed a high correlation with the Carstairs and Townsend indices, we think it allows more in depth analysis, when needed, and overcomes some of the limitations faced by between and within countries comparisons due to the low number and the nature of the variables than compose these well-known indices. Similar studies in other countries that allow usage of detailed socioeconomic information at BG levels would help assess the robustness of the procedure in other social contexts.

Fourthly, HC has no criteria regarding the size of the categories and so it can yield categories with very different sizes, which can be a limitation when linking their distribution with other attributes such as the prevalence of some health condition or of some exposure factor.

As a summary, a major strength of the procedure presented in this article is its versatility: it is not restricted to a particular set of data or type of study, and can be used for a large variety of contexts such as social epidemiology, environmental justice assessment, public health studies or urban and social planning. The application of this procedure on three large metropolitan areas shows high correlations with well-known indices like Townsend’s and Carstairs’, which appears to confirm that the created index represents the same socioeconomic notion. Although this procedure is more complicated than these other methods to create a SES index, the variables included in the final SES index allows a wider representation of the dimensions of SES, both to identify the best variables to distinguish BGs at the metropolitan area scale and to have better information on the particularities of the BGs. Then, it allows finer analysis of key determinants of health inequalities and reflection on local policies that would aim to cope with these inequalities. Another innovation in this study is the use of HC to constitute SES categories and compare them to the classically used quantiles. This approach allows having categories with more homogeneous compositions and which can consequently increase contrasts between them. Finally, we provide an R package able to reproduce the procedure easily.In conclusion, this procedure can be used to produce a SES index with a strong statistical basis and great scope for interpretation and relevance to public health bodies. The set of selected variables had a high proportion of common determinants of SES; they could also identify some features more specific to each area. Comparison of clustering methods showed that care should be taken to derive homogeneous categories.

## Abbreviations

BG: Census block group; HC: Hierarchical clustering; PCA: Principal component analysis; SES: Socio-economic status.

## Competing interests

The authors declare that they have no competing interests.

## Authors’ contributions

BL has contributed to the creation of the method, implemented the procedure, created the R package, performed and interpreted the application, drafted the article and conducted the literature review. JMM has contributed to the creation of the method, provided statistical rigor, contributed to the interpretation of the results and helped to draft the manuscript. CP has collected socioeconomic data and helped to draft the manuscript. WK has constructed some socioeconomic variables, filled the missing data for median income and helped to draft the manuscrip. NLM has given insight and expertise for the implementation and creation of the R package and helped to draft the manuscript. DZN guarantees quality assurance and helped to draft the manuscript. SD, head of Project Equit'Area research examining the role of environmental exposures on health inequalities, has followed up the general labor, has contributed to the definition of the method, interpretation of results, writing section and its finalization. All authors read and approved the final manuscript.

## Supplementary Material

Additional file 1 Base Socioeconomic Characteristics of the Three Study Urban Areas. Click here for file

Additional file 2 Principal component analysis and Hierarchical clustering. Click here for file

Additional file 3 Correlations and Contributions of Variables to the First Component and Variance Explained by the First Component, According to the Study Area. Click here for file

Additional file 4 Plot of city SES index vs. global index restricted to each city (housing census block groups only). Click here for file

Additional file 5 Plot of SES index vs. Carstairs’ index, according to the study area (housing census block groups only). Click here for file

Additional file 6 Plot of SES index vs. Townsend’s index, according to the study area (housing census block groups only). Click here for file

Additional file 7 Average Values of the Common Variables per Category Created With HC for the Global Analysis. Click here for file

Additional file 8 Maps of the socioeconomic index for Lille Metropole, in three categories by tiertiles or optimal thresholds. Click here for file

Additional file 9 Maps of the socioeconomic index for Grand Lyon, in three categories by tiertiles or optimal thresholds. Click here for file

Additional file 10 Maps of the socioeconomic index for Aix-Marseille urban area, in three categories by tiertiles or optimal thresholds. Click here for file
