# Supplementary material for: A statistical procedure to create a neighborhood socioeconomic index for health inequalities analysis
Source: Int J Equity Health. 2013 Mar 28;12:21. doi: 10.1186/1475-9276-12-21 (PMC3621558; doi:10.1186/1475-9276-12-21)
Supplement: Additional file 1 — Base Socioeconomic Characteristics of the Three Study Urban Areas. [file 1475-9276-12-21-S1.pdf]

Additional file 1. Base Socioeconomic Characteristics of the Three Study Urban Areas

	Lille Métropole	Grand Lyon	Aix-Marseille
People under the age of 25 (%)[b]	36.0 (32.8-40.6)	32.1 (28.8-35.8)	29.6 (25.7-33.7)
Single-parent families (%)[b]	13.9 (10.1-19.8)	13.9 (10.8-16.9)	17.5 (13.3-22.4)
Foreigners (%)[b]	3.9 (1.9-7.9)	6.6 (4.3-11.1)	3.5 (2.0-7.5)
Foreign immigrants since the last census (%)[b]	1.3 (0.7-2.2)	2.3 (1.2-3.6)	1.5 (0.9-3.0)
Unemployment rate (%)[b]	13.6 (9.2-19.9)	11.0 (8.3-15.0)	17.7 (13.2-25.3)
Self-employed (%)[b]	5.6 (4.0-8.3)	7.3 (5.3-10.5)	7.6 (5.3-11.4)
Unstable jobs (%)[b]	11.6 (9.1-14.7)	11.1 (9.0-13.6)	10.6 (8.5-12.4)
Steady jobs (%)[b]	67.4 (59.7-72.3)	68.2 (61.1-72.5)	61.7 (53.9-66.4)
Managers (%)[b]	9.7 (4.8-17.8)	13.4 (6.4-22.9)	9.3 (4.5-17.2)
Blue-collar workers (%)[b]	21.0 (13.7-28.3)	16.7 (10.2-25.1)	14.7 (8.9-20.0)
Median Income (€)[a, b]	21,165 (17,033-25,663)	22,656 (19,004-27,056)	20,318 (15,913-25,988)
People with no diploma (%)[b]	17.0 (10.6-23.6)	14.1 (9.9-21.7)	17.7 (12.5-25.3)
General or vocational maturity certificates (%)[b]	9.8 (8.3-11.9)	11.2 (9.4-12.7)	11.1 (8.7-12.9)
At least lower tertiary education (%)[b]	8.0 (5.7-10.3)	9.7 (6.4-12.2)	7.8 (5.1-10.2)
Individual houses (%)[b]	68.3 (33.0-88.5)	8.4 (1.1-37.9)	16.8 (3.3-60.3)
Multiple dwelling units (%)[b]	26.1 (7.0-62.3)	88.6 (57.5-96.6)	80.9 (36.3-94.0)
Non-owner-occupied (%)[b]	44.2 (25.9-69.7)	58.3 (39.9-72.6)	51.2 (35.2-68.7)
Subsidized housing (%)[b]	10.3 (0.6-29.0)	13.4 (2.2-34.7)	6.3 (0.0-27.3)
Houses with parking space (%)[b]	43.8 (26.5-64.4)	52.9 (34.0-72.7)	42.6 (22.3-67.2)
More than one person per room (%)[b]	6.0 (4.1-9.2)	7.1 (5.4-9.7)	7.7 (5.9-11.3)
Average number of people per room [a, b]	0.65 (0.61-0.72)	0.70 (0.66-0.76)	0.71 (0.67-0.76)
Without a car (%)[b]	24.1 (14.5-33.6)	23.1 (11.7-32.3)	21.9 (11.9-36.3)
With 2 or more cars (%)[b]	22.5 (14.7-37.2)	21.9 (14.5-34.7)	23.2 (12.6-36.2)

[a] Not a proportion ; [b] Median (Quartiles) among the census blocks of the metropolitan area

See Table 1 for precise definitions
