# Supplementary material for: A statistical procedure to create a neighborhood socioeconomic index for health inequalities analysis
Source: Int J Equity Health. 2013 Mar 28;12:21. doi: 10.1186/1475-9276-12-21 (PMC3621558; doi:10.1186/1475-9276-12-21)
Supplement: Additional file 2 — Principal component analysis and Hierarchical clustering. [file 1475-9276-12-21-S2.pdf]

Additional file 2: Principal component analysis and Hierarchical clustering

Principal component analysis [1–3]

Principal component analysis (PCA) is a data mining technique which aims to describe (highlight the similarity and dissimilarity between the statistical units and the correlations between the variables), summarize (determine a small number of new variables, uncorrelated linear combinations of the originals with maximal variance) and visualize the information contained in a data set.

Let X be a dataset with n rows representing the statistical units and p columns representing the variables (with the mean of each variable to zero). x_i^j will then be the value of the variable j for the unit i. Let s^j be the standard deviation of the jth variable. Let D be a diagonal matrix for the weights of the statistical units (frequently all the weights are equal to $1/n$).

The PCA studies first the statistical units in variables' space with the purpose to find a viewable graphical representation of these units, such as units with similar values will be represented by close points and units with very different values will be represented by distant points. To do so, a mathematical distance must be chosen. A very commonly used distance between the points i and i' is $d(i,i') = \sqrt{\sum_{j=1}^{p} \frac{1}{(s^j)^2} (x_i^j - x_{i'}^j)^2}$, but other distances can be used according to the purpose. The distance d is equivalent to set as metric on the space of the statistical units the diagonal matrix M such as $M_{j,j} = \frac{1}{(s^j)^2}$.

Then, the aim of the PCA is to visualize these points. Since the space's dimension (i.e. the number of variables) is in general important, it is necessary to project the points on an optimal sub-space of lower dimension, in order to have the most precise and faithful representation of the initial scatter plot. Hence, the PCA determines the sub-space F_r (with dimension r) which

maximizes inertia of the projections of the points on F_r with respect to the barycenter G (i.e. the weighted sum of the squared distances between G and the projections on F_r). To do so, an iterative process is used and leads to determine a set of orthonormal vectors $(\overrightarrow{u_1}, \ldots, \overrightarrow{u_k}, \ldots, \overrightarrow{u_r})$ which constitute a base of F_r, where $\overrightarrow{u_k}$ is the eigenvector of the matrix $X^T DXM$ corresponding to the kth largest eigenvalue, λ_k. The axis $(G, \overrightarrow{u_k})$ is called the kth *principal axis* and we obtain p principal axes, i.e the number of original variables. At this step, it is already possible to obtain the quality of representation and the contribution of each point on each principal axis.

Statistically, a principal axis represents a linear combination of the original variables called *principal component* and can be interpreted as the linear combination with maximal variance of the units (λ_k) given the constraint to be uncorrelated with the previous components. The ratio $\frac{\lambda_k}{\lambda_1 + \cdots + \lambda_p}$ can also be statistically interpreted as the percentage of variance explained by the kth factor. By construction, components are ordered from the one which explains the higher proportion of variance to the one which explains the less.

The interpretation of the PCA follows different steps. First, the number of axes to keep must be chosen. Secondly, thanks to the correlation circle created by the PCA, it is possible to visualize the correlations between the variables. Then, we interpret the components by studying the correlations between them and the variables. The last step is the interpretation of the points representing the units using their projections on the principal planes.

Hierarchical clustering [3,4]

Hierarchical clustering (HC) is an unsupervised method of clustering which creates a hierarchy of classes (i.e. clusters), frequently used after a PCA or others data mining techniques. Let I be a set of n elements ($I = \{1,2, \ldots, n\}$) represented by points in $\mathbb{R}^p$ and d a

distance between elements. The purpose is to find a partition in *r* classes which maximizes the between-classes inertia or, which is equivalent, which minimizes the within-classes inertia. This clustering criterion based on inertia allows creating classes homogeneous in their composition and heterogeneous between them. In practice, the search for a direct optimal solution requires generally too many computations and an approximation must be used. To do so, we use the algorithm of hierarchical clustering with a particular distance Δ between classes (based on *d*). This distance, called Ward's distance, is defined as following: Let I_1 and I_2 be two classes, p_1 and p_2 their respective weights, and G_1 and G_2 their respective barycenters, then the Ward's distance between classes I_1 and I_2 is $\Delta(I_1, I_2) = \frac{p_1 p_2}{p_1 + p_2} d^2(G_1, G_2)$.

The algorithm of hierarchical (ascending) clustering is then:

- Step 1: from the partition containing all the singletons $P_0 = \{\{1\}, \{2\}, \dots, \{n\}\}$ the distance Δ is computed for all the pairs of singletons. The classes $\{l\}$ and $\{m\}$with the minimum distance Δ are merged and then the partition with *(n-1)* elements obtained is $P_1 = \{\{l, m\}, \{1\}, \dots, \{n\}\}$.In others words, the two closest elements of *I* are merged in a single class while all the others remain singletons.
- …
- Step *r*: from the partition P_{r-1} with *(n-(r-1))* elements, the distance is computed between the elements of the partition. The classes with the minimal distance Δ are merged and the partition with *(n-r)* elements, P_r, is then created. The merge of these two classes is, by definition of the Ward's distance, the one which minimize the loss of between-classes inertia among all the others possible merging at this step.
- …
- Step *n-1*: the partition created is $P_{n-1} = \{I\}$

The HC presents as results a dendrogram (illustrating for each step of the algorithm the loss of between-classes inertia). The first step in the interpretation of the results is to choose the number of classes to keep. Generally, the partition that is chosen is the one preceding a strong decrease in the between classes inertia. However, others partitions can be chosen according to the purpose of the clustering. Once the number of classes determined, it is possible to interpret each class thanks to the comparison of the descriptive statistics of the variables between the class and the whole set.

References

1. Hastie T, Tibshirani R, Friedman J. Principal Components. In: *The elements of statistical learning*. 2nd ed. Springer; 2009:534-541.

2. Jolliffe IT. *Principal component analysis*. 2nd ed. New York: Springer; 2002.

3. Lebart L, Morineau A, Warwick KM. *Multivariate descriptive statistical analysis : correspondence analysis and related techniques for large matrices*. New York: Wiley; 1984.

4. Hastie T, Tibshirani R, Friedman J. Hierarchical Clustering. In: *The elements of statistical learning*. 2nd ed. Springer; 2009:520-528.
