# Supplementary material for: A statistical procedure to create a neighborhood socioeconomic index for health inequalities analysis
Source: Int J Equity Health. 2013 Mar 28;12:21. doi: 10.1186/1475-9276-12-21 (PMC3621558; doi:10.1186/1475-9276-12-21)
Supplement: Additional file 3 — Correlations and Contributions of Variables to the First Component and Variance Explained by the First Component, According to the Study Area. [file 1475-9276-12-21-S3.pdf]

Additional file 3. Correlations and Contributions of Variables to the First Component and Variance Explained by the First Component, According to the Study Area

Variables	Lille Métropole		Grand Lyon		Marseille urban unit		Global	
	Coord [a]	*Ctr* [b]	*Coord* [a]	*Ctr* [b]	*Coord* [a]	*Ctr* [b]	*Coord* [a]	*Ctr* [b]
SINGLE-PARENT FAMILIES	0.86	5.79	0.79	5.44	0.86	6.38	0.83	6.29
FOREIGNERS	0.82	5.33	0.87	6.59	0.79	5.51	0.78	5.66
FOREIGN IMMIGRANTS (SINCE THE LAST CENSUS)	0.70	3.88	0.67	3.84	0.65	3.67	0.62	3.59
SELF-EMPLOYED	-0.61	2.89	-0.70	4.22	-0.63	3.45	-0.62	3.57
UNSTABLE JOBS	0.88	6.14	0.79	5.45	0.62	3.32	0.73	4.91
STEADY JOBS	-0.89	6.16	-0.76	5.03	-0.85	6.29	-0.83	6.29
MEDIAN INCOME	-0.71	3.97	-0.78	5.27	-0.86	6.48	-0.80	5.83
NO DIPLOMA	0.74	4.29	0.84	6.14	0.81	5.69	0.83	6.33
BASIC OR INTERMEDIATE GENERAL OR VOCATION QUALIFICATIONS	-0.71	3.98	-0.79	5.43	-0.72	4.52	-0.74	5.06
GENERAL OR VOCATIONAL MATURITY CERTIFICATES	-0.64	3.26	-0.75	4.88	-0.75	4.95	-0.74	5.08
NON-OWNER-OCCUPIED IN THE MAIN RESIDENCES	0.85	5.61	0.77	5.11	0.83	6.01	0.78	5.62
MAIN RESIDENCES WITH MORE THAN ONE PERSON PER ROOM	0.81	5.16	0.83	5.91	0.74	4.73	0.79	5.74
AVERAGE NUMBER OF PEOPLE PER ROOM	0.85	5.66	0.78	5.24	0.73	4.64	0.76	5.26
HOUSEHOLDS WITHOUT A CAR	0.90	6.30	0.66	3.80	0.84	6.13	0.79	5.79
HOUSEHOLD WITH 2 OR MORE CARS	-0.86	5.77	-0.67	3.92	-0.80	5.55	-0.76	5.27
Under age of 25	0.65	3.28	-	-	-	-	-	-
Unemployed for more than 1 year	0.87	5.97	-	-	-	-	-	-
Individual house	-0.69	3.69	-	-	-0.65	3.66	-	-
Multiple dwelling units	0.67	3.51	-	-	0.63	3.50	-	-
Subsidized housing	0.75	4.38	0.80	5.50	-	-	0.67	4.13
Parking space	-0.80	4.98	-0.59	3.01	-0.75	4.87	-0.71	4.66
Unemployed	-	-	0.93	7.48	0.92	7.44	0.90	7.38
Managers	-	-	-0.65	3.64	-0.61	3.20	-0.62	3.55
Blue-collar workers	-	-	0.69	4.11	-	-	-	-
Contribution of the common variables to the 1st component	80.16%		83.75%		77.62%		87.67%	
Variance explained by the first component	60.73%		57.79%		57.29%		57.22%	

[a] Coordinate of the variable on the first axis, i.e. correlation coefficient between the variable and the socio-economic index

[b] Contribution of the variable to the first component (in %)

UPPERCASE : variables selected commonly for each metropolitan areas and global analysis
