# Supplementary material for: A statistical procedure to create a neighborhood socioeconomic index for health inequalities analysis
Source: Int J Equity Health. 2013 Mar 28;12:21. doi: 10.1186/1475-9276-12-21 (PMC3621558; doi:10.1186/1475-9276-12-21)
Supplement: Additional file 4 — Plot of city SES index vs. global index restricted to each city (housing census block groups only). [file 1475-9276-12-21-S4.pdf]

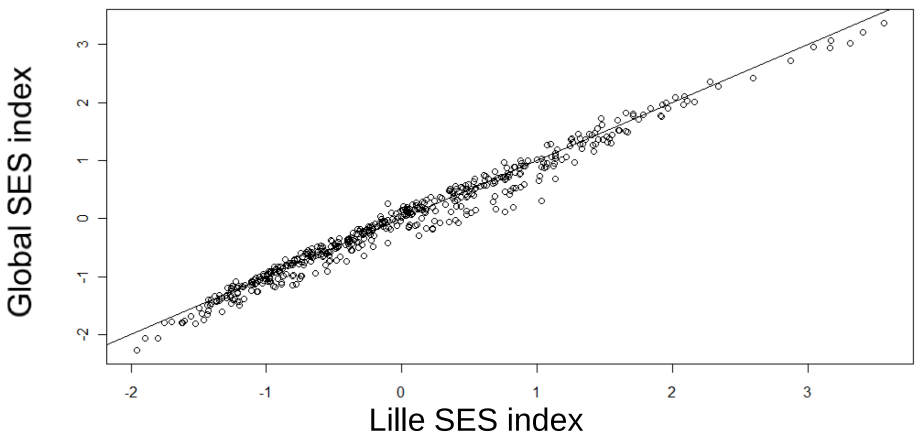

A. Lille

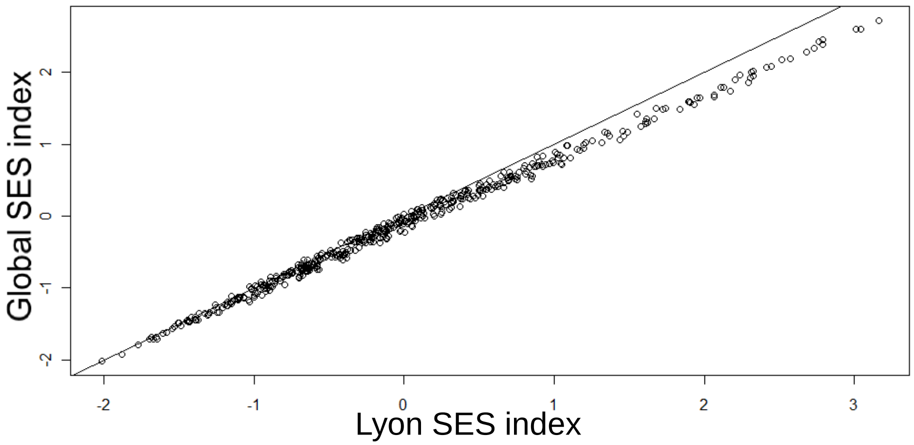

B. Lyon

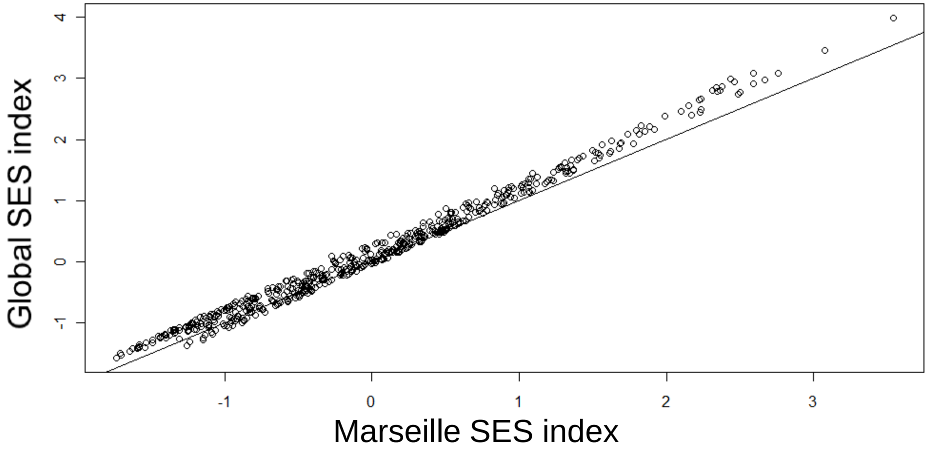

C. Marseille

Additional file 4. Plot of city SES indices vs. global index restricted to each city (housing census blocks only).
