# Supplementary material for: A statistical procedure to create a neighborhood socioeconomic index for health inequalities analysis
Source: Int J Equity Health. 2013 Mar 28;12:21. doi: 10.1186/1475-9276-12-21 (PMC3621558; doi:10.1186/1475-9276-12-21)
Supplement: Additional file 5 — Plot of SES index vs. Carstairs’ index, according to the study area (housing census block groups only). [file 1475-9276-12-21-S5.pdf]

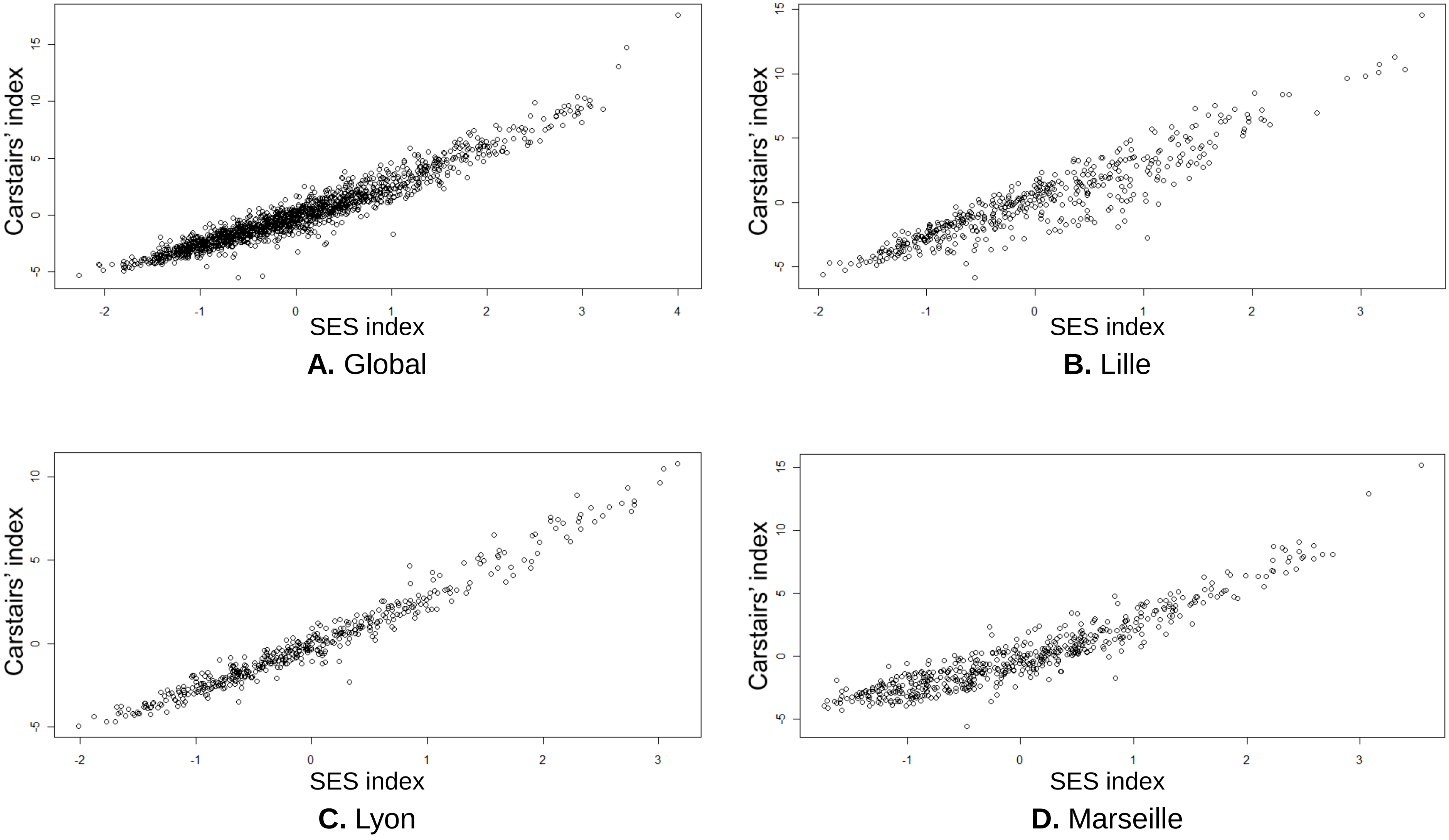

Additional file 5. Plot of SES index vs. Carstairs' index, according to the study area (housing census blocks only).
