# Supplementary material for: A statistical procedure to create a neighborhood socioeconomic index for health inequalities analysis
Source: Int J Equity Health. 2013 Mar 28;12:21. doi: 10.1186/1475-9276-12-21 (PMC3621558; doi:10.1186/1475-9276-12-21)
Supplement: Additional file 6 — Plot of SES index vs. Townsend’s index, according to the study area (housing census block groups only). [file 1475-9276-12-21-S6.pdf]

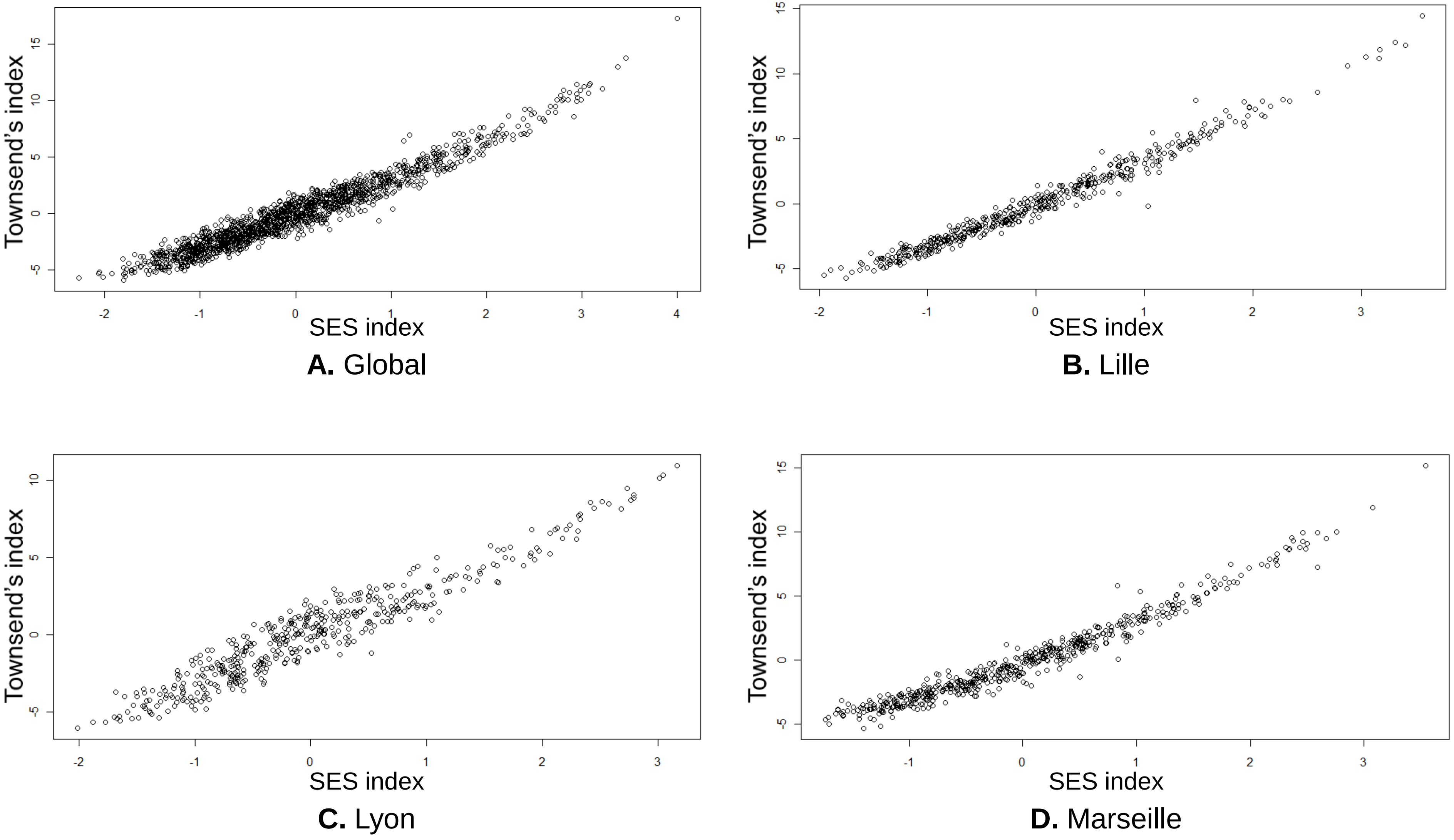

Additional file 6. Plot of SES index vs. Townsend's index, according to the study area (housing census blocks only).
