# Supplementary material for: A statistical procedure to create a neighborhood socioeconomic index for health inequalities analysis
Source: Int J Equity Health. 2013 Mar 28;12:21. doi: 10.1186/1475-9276-12-21 (PMC3621558; doi:10.1186/1475-9276-12-21)
Supplement: Additional file 7 — Average Values of the Common Variables per Category Created With HC for the Global Analysis. [file 1475-9276-12-21-S7.pdf]

Additional file 7. Average Values of the Common Variables per Category Created With HC for the Global Analysis

Variables	Category 1 (lowest index values)	Category 2	Category 3 (highest index values)
SINGLE-PARENT FAMILIES	11.67%	17.56%	26.07%
FOREIGNERS	2.96%	7.02%	17.49%
FOREIGN IMMIGRANTS (SINCE THE LAST CENSUS)	1.32%	2.38%	5.29%
SELF-EMPLOYED	10.52%	6.48%	4.04%
UNSTABLE JOBS	8.92%	12.70%	14.95%
STEADY JOBS	69.88%	62.91%	45.01%
MEDIAN INCOME	27,448 €	19,832 €	13,517 €
NO DIPLOMA	12.24%	19.17%	33.90%
BASIC OR INTERMEDIATE GENERAL OR VOCATION QUALIFICATIONS	12.46%	10.00%	7.17%
GENERAL OR VOCATIONAL MATURITY CERTIFICATES	10.58%	7.71%	3.94%
NON-OWNER-OCCUPIED IN THE MAIN RESIDENCES	33.72%	60.60%	80.38%
MAIN RESIDENCES WITH MORE THAN ONE PERSON PER ROOM	5.62%	8.31%	18.86%
AVERAGE NUMBER OF PEOPLE PER ROOM	0.65 person/room	0.71 person/room	0.83 person/room
HOUSEHOLDS WITHOUT A CAR	13.00%	30.40%	42.76%
HOUSEHOLD WITH 2 OR MORE CARS	39.30%	17.89%	11.27%
Subsidized housing	7.18%	25.08%	60.15%
Parking space	66.48%	34.91%	22.57%
Unemployed	10.43%	17.67%	35.72%
Managers	18.41%	11.37%	3.19%

UPPERCASE : variables selected commonly for each metropolitan areas and global analysis
