# Supplementary material for: A statistical procedure to create a neighborhood socioeconomic index for health inequalities analysis
Source: Int J Equity Health. 2013 Mar 28;12:21. doi: 10.1186/1475-9276-12-21 (PMC3621558; doi:10.1186/1475-9276-12-21)

Additional file 8. Maps of the socioeconomic index for Lille Metropole, in three categories by tiertiles or optimal thresholds

Neighborhood socioeconomic Index in 3 categories by optimal thresholds for Lille Metropole

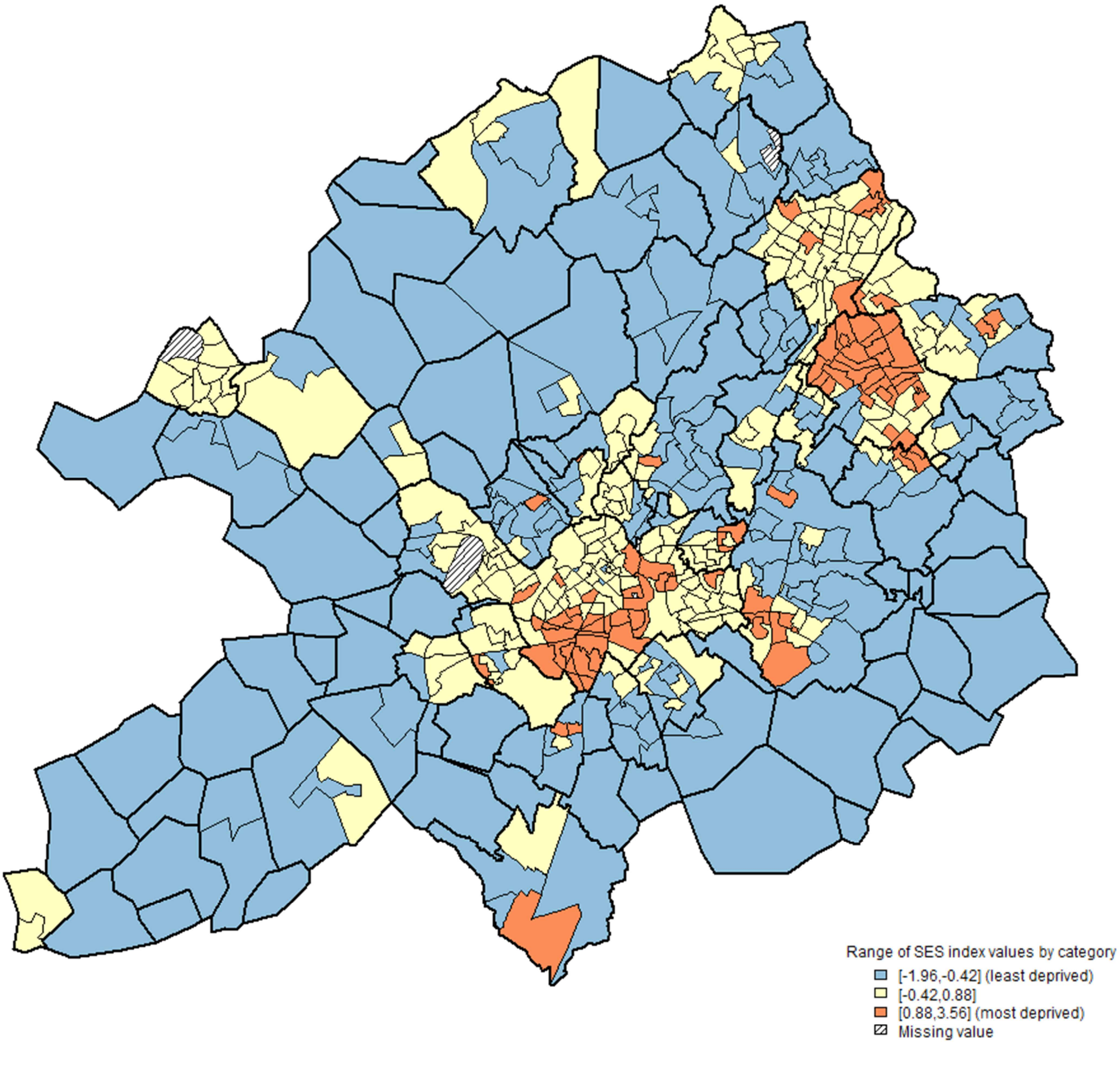

Neighborhood socioeconomic Index in 3 categories by tiertiles for Lille Metropole

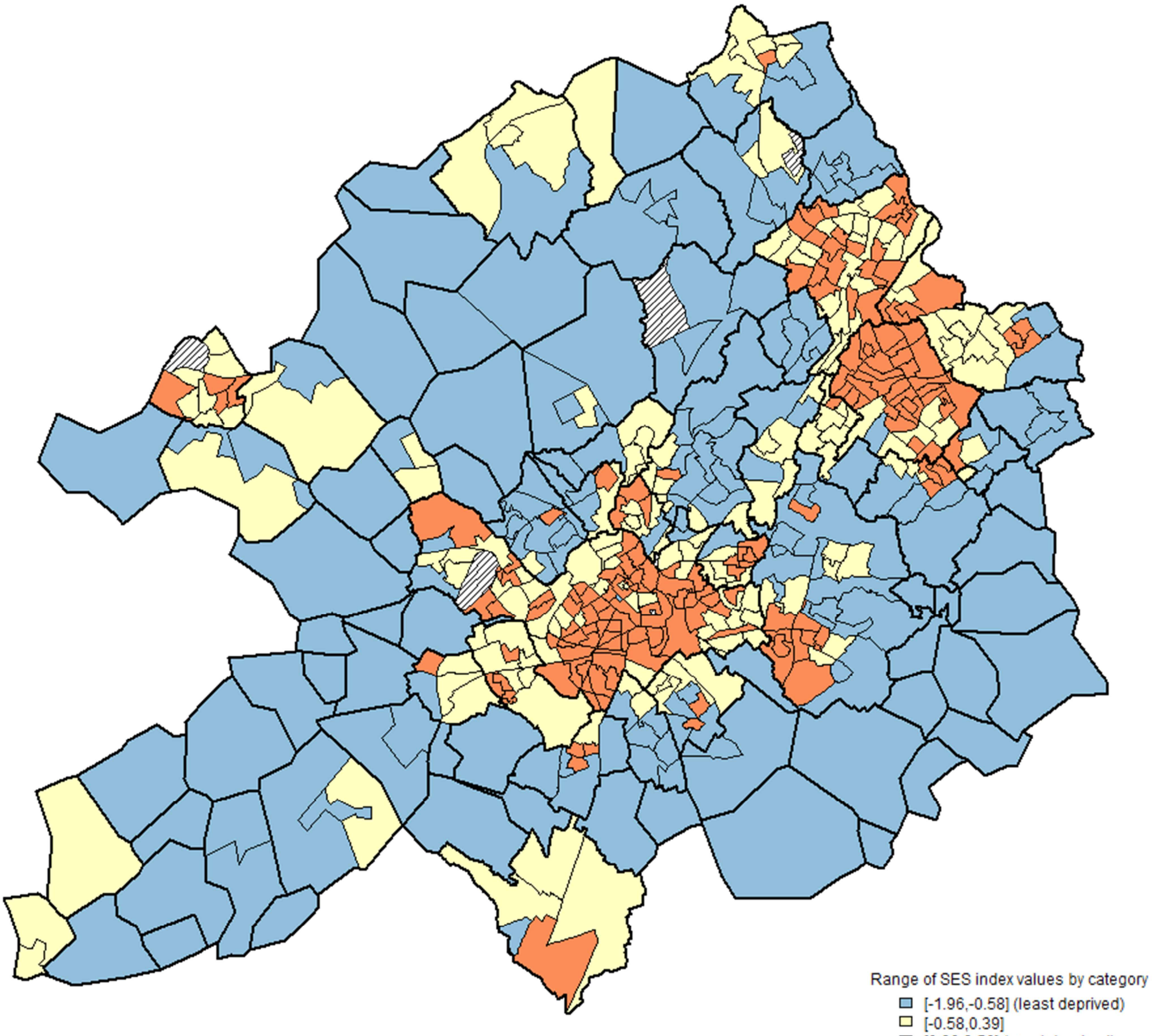

Supplement: Additional file 8 — Maps of the socioeconomic index for Lille Metropole, in three categories by tiertiles or optimal thresholds. [file 1475-9276-12-21-S8.pdf]
