# Supplementary material for: A statistical procedure to create a neighborhood socioeconomic index for health inequalities analysis
Source: Int J Equity Health. 2013 Mar 28;12:21. doi: 10.1186/1475-9276-12-21 (PMC3621558; doi:10.1186/1475-9276-12-21)

Additional file 9. Maps of the socioeconomic index for Grand Lyon, in three categories by tiertiles or optimal thresholds

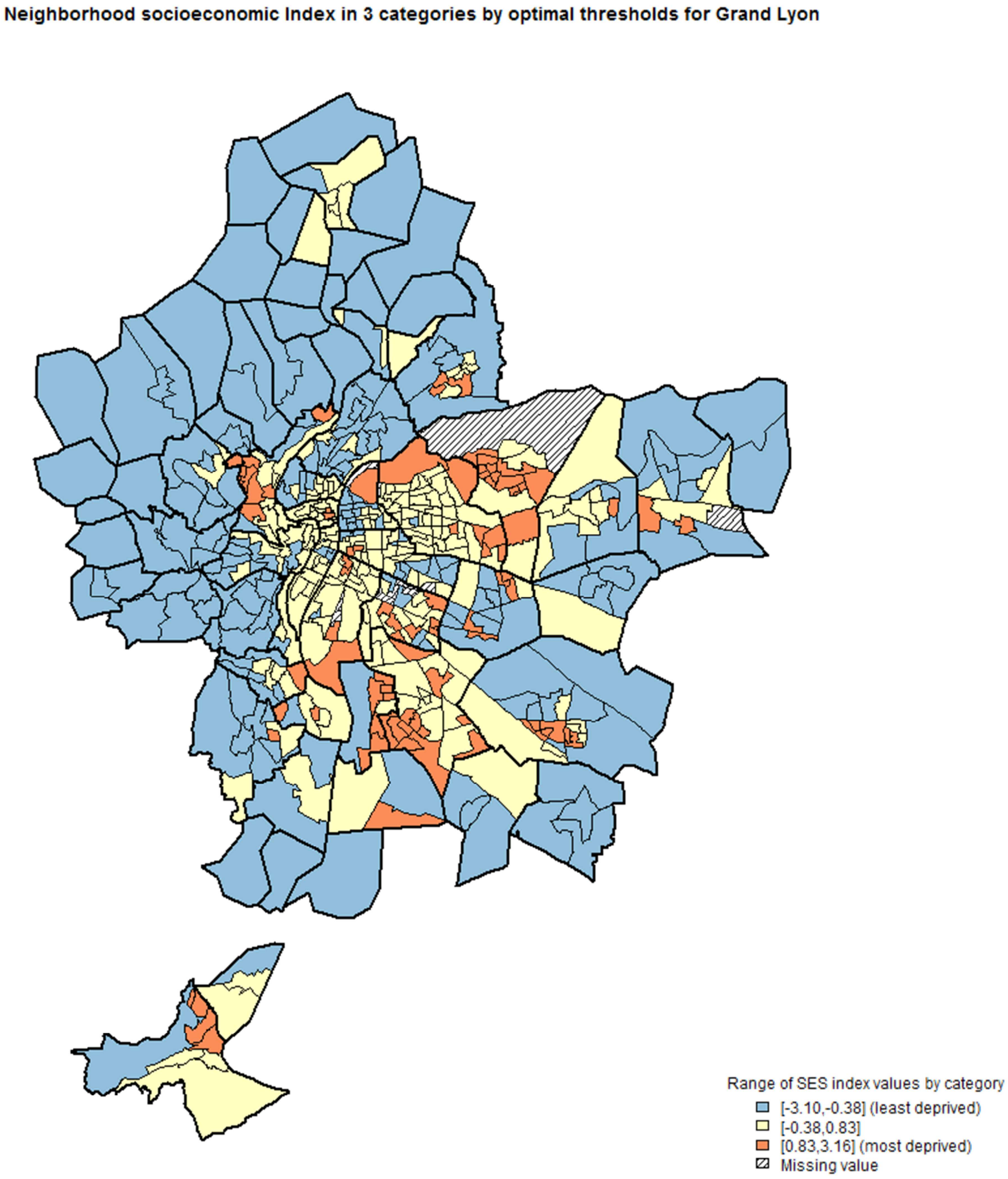

Neighborhood socioeconomic Index in 3 categories by tiertiles for Grand Lyon

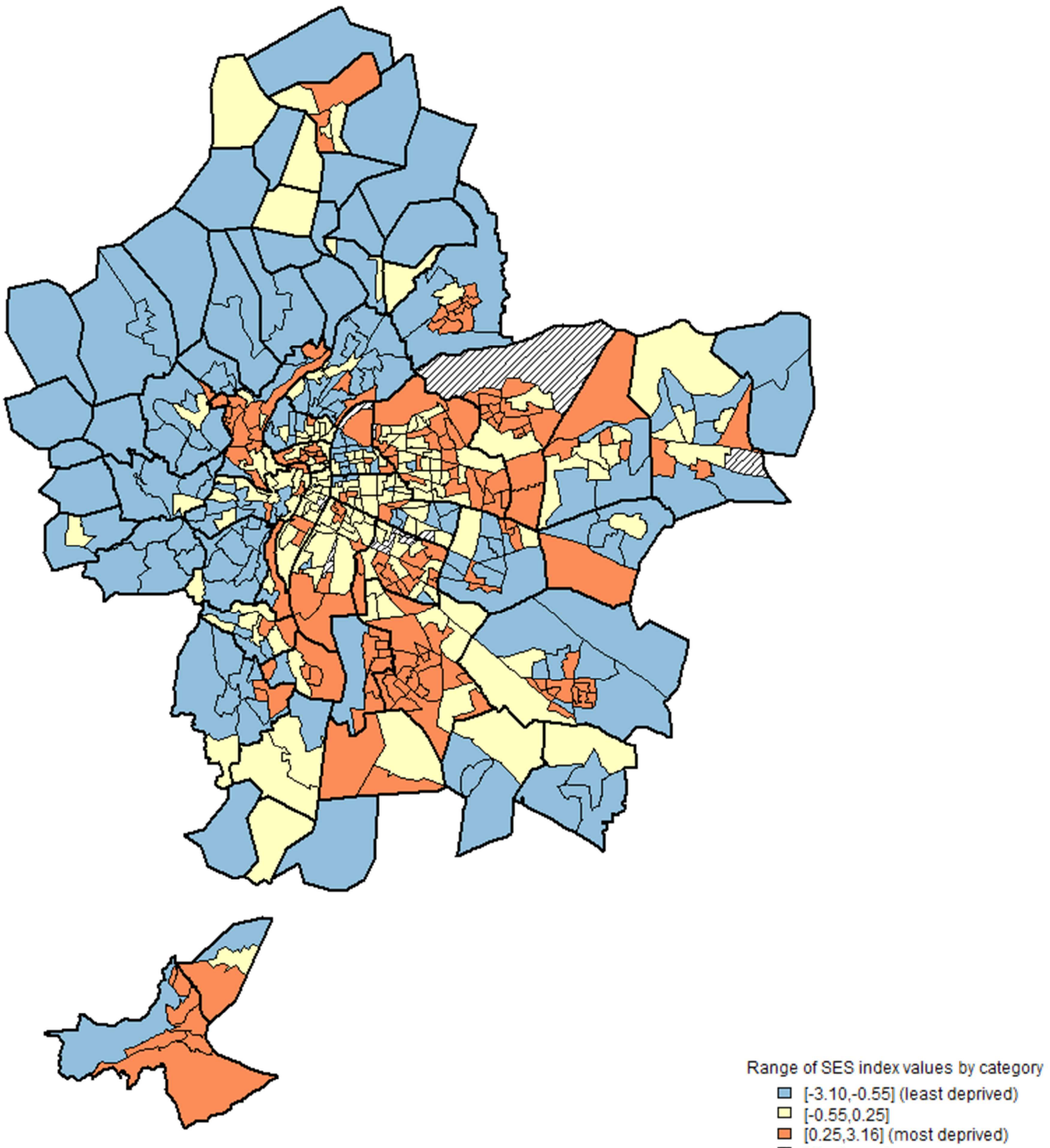

Supplement: Additional file 9 — Maps of the socioeconomic index for Grand Lyon, in three categories by tiertiles or optimal thresholds. [file 1475-9276-12-21-S9.pdf]
