# Supplementary material for: A statistical procedure to create a neighborhood socioeconomic index for health inequalities analysis
Source: Int J Equity Health. 2013 Mar 28;12:21. doi: 10.1186/1475-9276-12-21 (PMC3621558; doi:10.1186/1475-9276-12-21)

**Additional file 10. Maps of the socioeconomic index for Aix-Marseille urban area, in three categories by tiertiles or optimal thresholds**

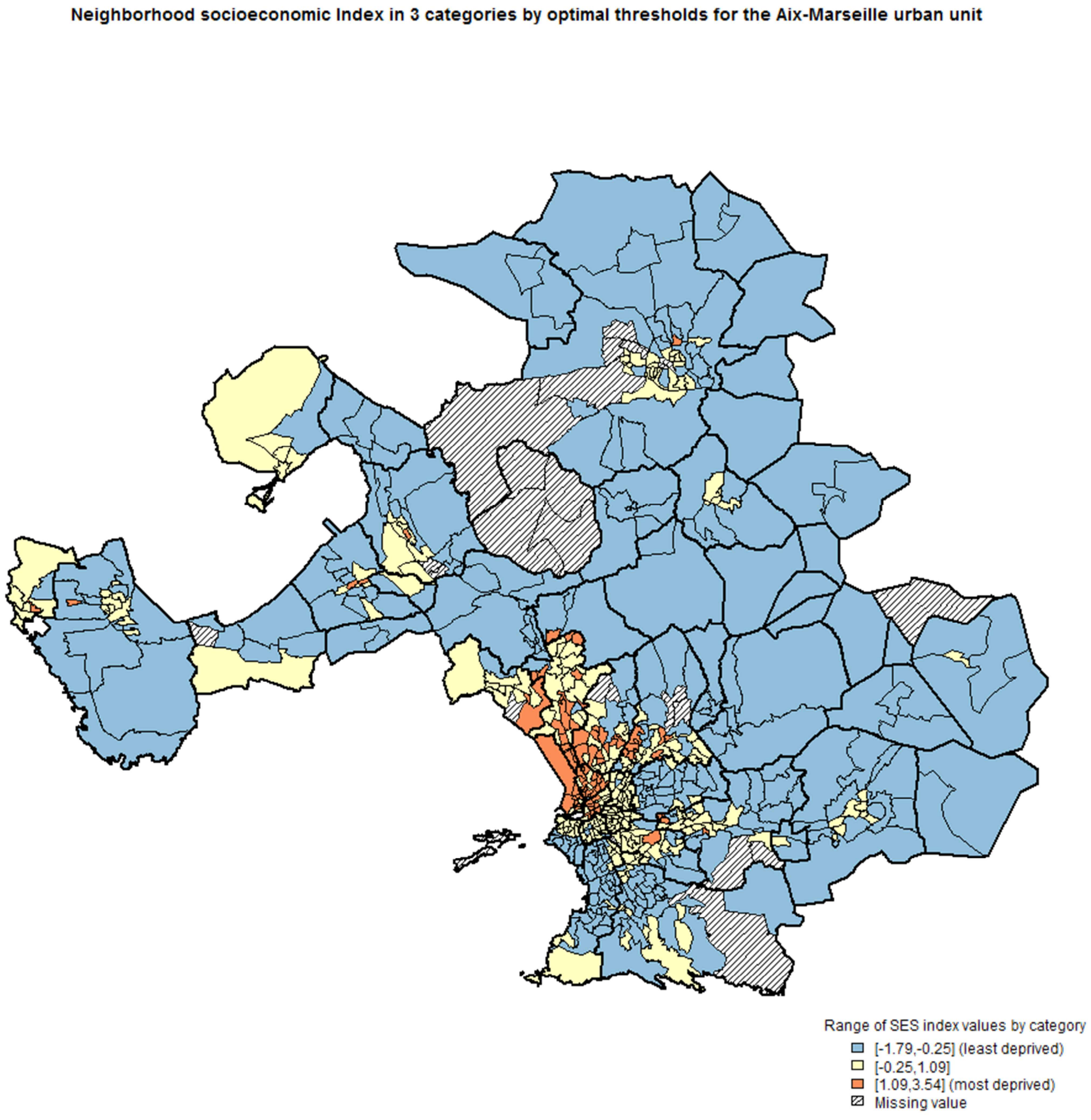

**Neighborhood socioeconomic Index in 3 categories by tiertiles for the Aix-Marseille urban unit**

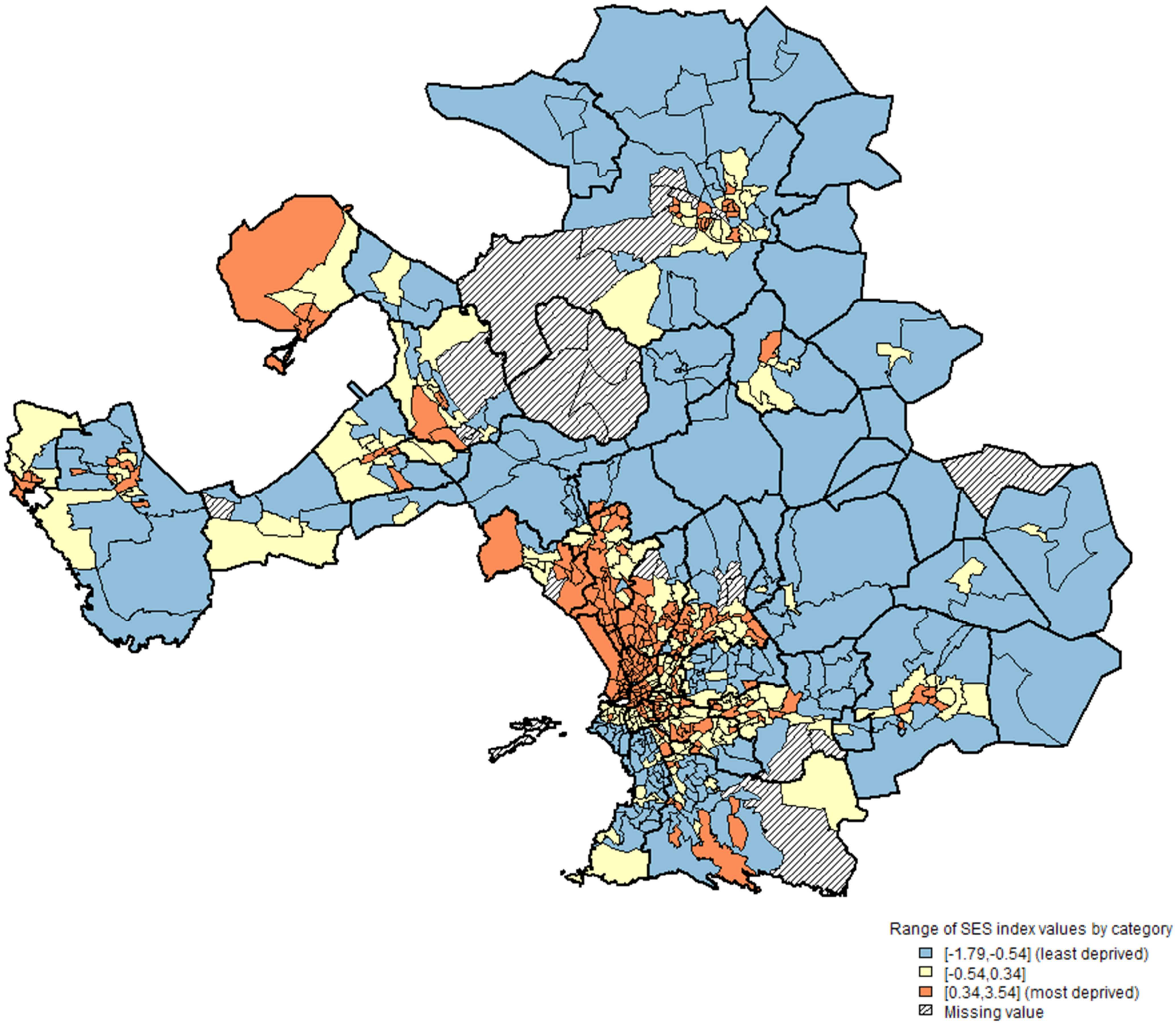

Supplement: Additional file 10 — Maps of the socioeconomic index for Aix-Marseille urban area, in three categories by tiertiles or optimal thresholds. [file 1475-9276-12-21-S10.pdf]
